# An efficient resource utilization scheme within PMIPv6 protocol for urban vehicular networks

**DOI:** 10.1371/journal.pone.0212490

**Published:** 2019-03-07

**Authors:** Safwan M. Ghaleb, Shamala Subramaniam, Zuriati Ahmad Zukarnain, Abdullah Muhammed, Mukhtar Ghaleb

**Affiliations:** 1 Department of Communication Technology and Network, Universiti Putra Malaysia, Serdang, Selangor D.E., Malaysia; 2 Sports Academy, Universiti Putra Malaysia, Serdang, Selangor D.E, Malaysia; 3 University of Bisha, Al-Namas branch, Al-Namas, Saudi Arabia; 4 Faculty of Computer Science and Information Technology, Sana’a University, Sana’a, Yemen; University of British Columbia, CANADA

## Abstract

Recently, the mobility management of urban vehicular networks has become great challenges for researchers due to its unique mobility requirements imposed by mobile users when accessing different services in a random fashion. To provide a ubiquitous Internet and seamless connectivity, the Internet Engineering Task Force (IETF) has proposed a Proxy Mobile IPv6 (PMIPv6) protocol. This is meant to address the signaling of the mobility transparent to the Mobile Node (MN) and also guarantee session continuity while the MN is in motion. However, performing a handoff by tens of thousands of MNs may harm the performance of the system significantly due to the high signaling overhead and the insufficient utilization of so-called Binding Cash Entry (BCE) at the Local Mobility Anchor (LMA). To address these issues, we propose an efficient scheme within the PMIPv6 protocol, named AE-PMIPv6 scheme, to effectively utilize the BCE at the LMA. This is primarily achieved by merging the BCEs of the MNs, thus, reducing the signaling overhead. Better utilization of the BCEs has been attained by employing virtual addresses and addressing pool mechanisms for the purpose of binding information of the MNs that are moving together towards the same network at a specific time, during their handoff process. Results obtained from our simulation demonstrates the superiority of AE-PMIPv6 scheme over E-PMIPv6 scheme. The AE-PMIPv6 succeeds in minimizing the signaling overhead, reduces the handover time and at the same time efficiently utilize the buffer resources.

## Introduction

Recently, research works have shown an increasing interest in the mobility management protocols, especially in urban vehicular networks. This is due to the rapid growth of mobile users who are eager to enjoy the Internet multimedia services (e.g., video, audio, web browsing, and uploading or downloading files) as they roam between different networks. Hence, enhancing the user experience of such a service requires the design of an effective handoff scheme, which has been proved to be challenging issue within the context of the urban vehicular networks is. This is due to the high vehicle velocity, density and the non-deterministic patterns of users’ mobility. In order to provide a ubiquitous Internet, the Quality of Service (QoS) should meet the user mobile requirements. Subsequently, the vehicles in cities have to support the seamless handoff by minimizing the signaling cost, packet loss, handover latency, etc.

In fact, the scenario of wireless connectivity in urban vehicular networks is different from the scenario of vehicular networks on highways. In the latter, the mobile users directly connect to a Fixed Roadside Unit (RSU) to obtain the Internet wireless services. In the former, the public transportation such as buses and trains may be equipped with Mobile Router (MR) to provide wireless connections to the mobile users. Hence, the mobility becomes a complex issue due to the frequent change of mobile users’ points of attachment (e.g., when the mobile user disconnects from RSU/MR to connect to another MR/RSU), which greatly degrades the overall system performance. Consequently, the handoff process in the mobility management protocols should be performed efficiently when the mobile users cross different networks. This is performed by designing an efficient system that considers the packet loss, handoff latency, signaling overhead, etc., during the handoff operations.

The Mobile IPv6 (MIPv6) protocol is developed by the IETF [1] to support the global mobility for the Mobile Nodes (MNs). Protocols such as Hierarchical MIPv6 (HMIPv6) [2], a Fast Handover for Hierarchical MIPv6 (FHMIPv6) [3], Fast-Handovers for Mobile IPv6 (FMIPv6) [4], etc., are released by the IETF to provide a seamless handover. However, the MNs in these protocols must involve in the mobility-related signaling. Moreover, these protocols support the mobility for each MN individually. In order to enhance the mobility management, PMIPv6 localized management protocol has been standardized by the IETF [5]. This is achieved by exempting the MN from any signaling that is related to the mobility management. Another network-based protocol called Fast Handover for PMIPv6 (PFMIPv6) is proposed in order to provide a seamless connection [6]. This protocol has two scenarios: proactive and reactive. In the proactive mode, the PFMIPv6 protocol has to setup a tunnel between the Previous Mobility Access Gateway (PMAG) and the New MAG(NMAG) in prior to prevent the packet loss. The tunneling setup is performed by exchanging the Handover Initiate (HI) and Handover Acknowledge (HAck) messages [7]. Nevertheless, both the PMIPv6 and PFMIPv6 protocols still manage the MNs’ handoff process individually.

Network Mobility Basic Support (NEMO-BS) protocol [8] has been proposed by the IETF to support the mobility management for an entire network. Due to its perceived efficiency, several Intelligent Transport Systems (ITS) projects (such as Cooperative Vehicle Infrastructure Systems (CVIS) [9], GeoNetworking [10], Communication Architecture for Land Mobile (CALM) [11]) consider the NEMO-BS protocol as an important part of their infrastructure for the mobility management. Despite of the gain achieved by grouping the MNs to enhancing the mobility management protocols, the handoff process of the MR in the NEMO-BS protocol is similar to that of the MIPv6 protocol. This degrades the system performance due to the long delay handoff, in addition to the lack of mobility support for the MNs that are moving between different networks individually. Consequently, the Efficient Proxy Mobile IPv6 (E-PMIPv6)-based handoff scheme [12]has been proposed to enhance the mobility management by exempting the MNs from any mobility-related signaling in urban vehicular networks. This is performed by enabling the individual MNs to connect to the wireless network from either fixed point or MR seamlessly. Furthermore, the E-PMIPv6 scheme introduces one Home Network Prefix (HNP) for several MNs within the same network, which occupies one BCE at the LMA. In the other protocols, the BCE must be created and assigned to every MN connected to the network, which consumes the buffer resources which has been alleviated in the E-PMIPv6 operations. In spite of the improvements that have been made by the E-PMIPv6 scheme, the E-PMIPv6 and NEMO-BS protocol depend on the MR to enhance the mobility managements and utilize the BCEs at the LMA. Hence, some public and private transportation may not be equipped with MR, which makes the prior work scenarios insufficient due to their dependencies on the MRs.

In this paper, we proposed the AE-PMIPv6 scheme to solve the issues related to the E-PMIPv6 scheme through the introduction of the notion of virtual addresses mechanism and utilizing the addressing pool mechanism. The virtual addresses mechanism is used to merge the MNs’ binding information allowing for a better utilization of the BCEs at the LMA, while the addressing pool is used to keep the addresses in the MAG’s pool that are ordered in advance. In the AE-PMIPv6 scheme, a BCE is used to maintain the associated MNs and the MRs information related to their arrival time and their previous networks, which differs from the E-PMIPv6 scheme where a BCE must be created for every MN changing its MAG, thus, increasing the buffer cost. In addition, the E-PMIPv6 scheme must also create a BCE for every MR at the LMA domain in order to keep the information of the connected MR and its members.

Accordingly, this paper proposes several contributions demonstrated as follows:

1. An efficient utilization of the BCEs at the LMA (buffer resource utilization) via merging the BCEs of several MNs that reside on the same network in one BCE, where LMA is responsible for handling tens of thousands of MNs connections.
2. An efficient mode for the MNs registration is proposed to reduce the signaling cost and increase the scalability of the network by employing the virtual addressing scheme.
3. An extensive simulation to validate the proposed AE-PMIPv6 scheme.

The rest of this paper is organized as follows: Section 2 reviews the related work and briefly describes the PMIPv6 protocol. Section 3 presents the system model. Section 4 explicitly illustrates the AE-PMIPv6 scheme. Section 5 introduces a detailed description of the simulation environment and the obtained results. Section 6 presents the concluding remarks.

## Related studies

The PMIPv6 has been introduced by the IETF to support the network-based mobility in the localized domain relieving the MNs from any handoff signaling. The PMIPv6 standard has introduced two new functional entities, namely LMA and MAG. The function of the LMA entity is to create the HNPs that makes the MNs transparent from any mobility signaling and also forward the packets that come from the connected MNs to the Corresponding Nodes (CNs). The MAG entity usually functions as an intermediate access router to forward the data packets between the MNs and the LMA as well as to emulate the HNP by sending a Router Advertisement message (RA) to the MN during the registration phase. The LMA, after allocating the HNPs for the registered MNs, maintains their information in the BCEs. The handoff process is triggered when the MN moves from its PMAG to its NMAG. Upon triggering the handoff process, the LMA updates its BCE by changing the Identifier (ID) of the PMAG in the MAG field with the NMAG’s ID. This is done to keep a track of the new linked MAG. PMIPv6 exempts the MNs from any handoff signaling by delegating the mobility burdens to the Internet Protocol (IP) network entities (MAG and LMA). This is done to provide mobility management without changing the MNs’ IP addresses during the handoff process. However, the PMIPv6 still suffers from handover latency, single point of failure and packet loss, in addition to the lack of support of the simultaneous mobility of multiple MNs [13].

In order to improve the mobility support and mitigate the issues of PMIPv6, Fast Handover for Proxy Mobile IPv6 based on 802.11 Networks (PFMIPv6) is proposed by [14]. To achieve these, the HNPs and authentication information of the MNs are exchanged between the neighboring routers. In the PFMIPv6 protocol, the PMAG of the MN establishes a pre-tunnel with the MN’s NMAG upon receiving the link layer report from the handoff MN. This tunnel is used to forward the buffered packets in the PMAG to the NMAG when the MN starts its handoff process to limit the packet loss. However, a high packet loss is still experienced in this protocol as a result of wrong prediction of MNs’ movement. Researchers in the field of vehicular network propose the Early Binding Update Registration in PMIPv6 (EBRPMIPv6) to enable the mobility in vehicular networks [15]. The main aim of this scheme is to shorten the handover time. The neighboring MAG table enables the PMAG to identify the exact NMAG in prior, which is done by employing the information obtained from the Global Positioning System (GPS) and the MNs’ movement direction. Another similar work proposed in [16] uses the GPS to identify the NMAG in order to improve the handoff. When the NMAG is identified, a bi-directional tunnel is established between the PMAG and the NMAG to deliver the buffered packet. Vehicular IP in Wireless Access in Vehicular Environments (VIP-WAVE) is proposed to adopt the PMIPv6 based localized mobility management in order to support IP services in 802.11p/WAVE networks. The works in [14–18] are presented to enhance the mobility management for the individual MNs by either reducing the handoff time or packet loss. The work in [17], proposes a mobility handover scheme MHVA for IPv6-based vehicular ad hoc networks to achieve a better QoS with regards to the handover latency by performing the operations of the network layer handoff before the operations of the link layer handoff.

The main issue with the aforementioned protocols is that the mobility management is supported only for one MN at a time. Consequently, the overall system performance degrades due to the mobility management for a group of MNs is not considered. To solve this issue, NEMO-BS protocol is standardized by the IETF to provide a NEMO for the MNs that move in a group [8]. An MR is the new entity, which takes the responsibility for performing the mobility management for all MNs attached to it as well as providing wireless connections to its member MNs. In the registration phase, the MR requires a Home of Address (HoA) from the home network to configure its IP address, while the Care of Address (CoA) is required if the MR moves to another access network. Then, the MR exchanges a binding update message with the HoA to bind the new CoA in the BCE at the Home Agent (HA). Once the binding occurs, a bi-directional tunnel is established between the MR and the HA. Consequently, the process of the handoff is carried out by the MR for a group of MNs instead of performing it independently by each MN. This reduces the signaling overhead significantly in the system. However, the handoff process in NEMO-BS protocol is performed in a way similar to that of the MIPv6, which results in the same long handoff delay. This is due to performing Duplicate Address Detection (DAD) after acquiring CoA. Moreover, the MR needs to exchange a binding update message with the HA every time MR changes its location contributing to the increased handoff latency.

To tackle the issue related to the NEMO-BS protocol, PMIPv6-based NEMO (P-NEMO) is developed to provide the PMIPv6 with capacity of network mobility [19]. This is performed to shield the handoff signaling from the entire mobile network in the intelligent transportation system. The P-NEMO itself has been extended in order to introduce a new protocol, named Fast P-NEMO (FP-NEMO) seeking to improve the mobility support for an entire mobile network [19]. To achieve this, a bi-directional tunnel is established in advance between the PMAG and the NMAG in the handoff procedure to forward the packet coming from the uplink or downlink link. However, the P-NEMO and FP-NEMO only support the mobility management for the mobile network [19]. Hence, an Enhanced FMIPv6 based on NEMO scheme (EfNEMO) is introduced to support the mobility management for a mobile network [20]. A binding update is temporarily conducted by the MR to register the new CoA before starting the link layer (L2) handoff. Accordingly, the path between the HA and the NMAG are used to forward the packets to the MNs. Thus, and the packet tunneling between the PMAG and the NMAG will be eliminated.

**Fig 1:**
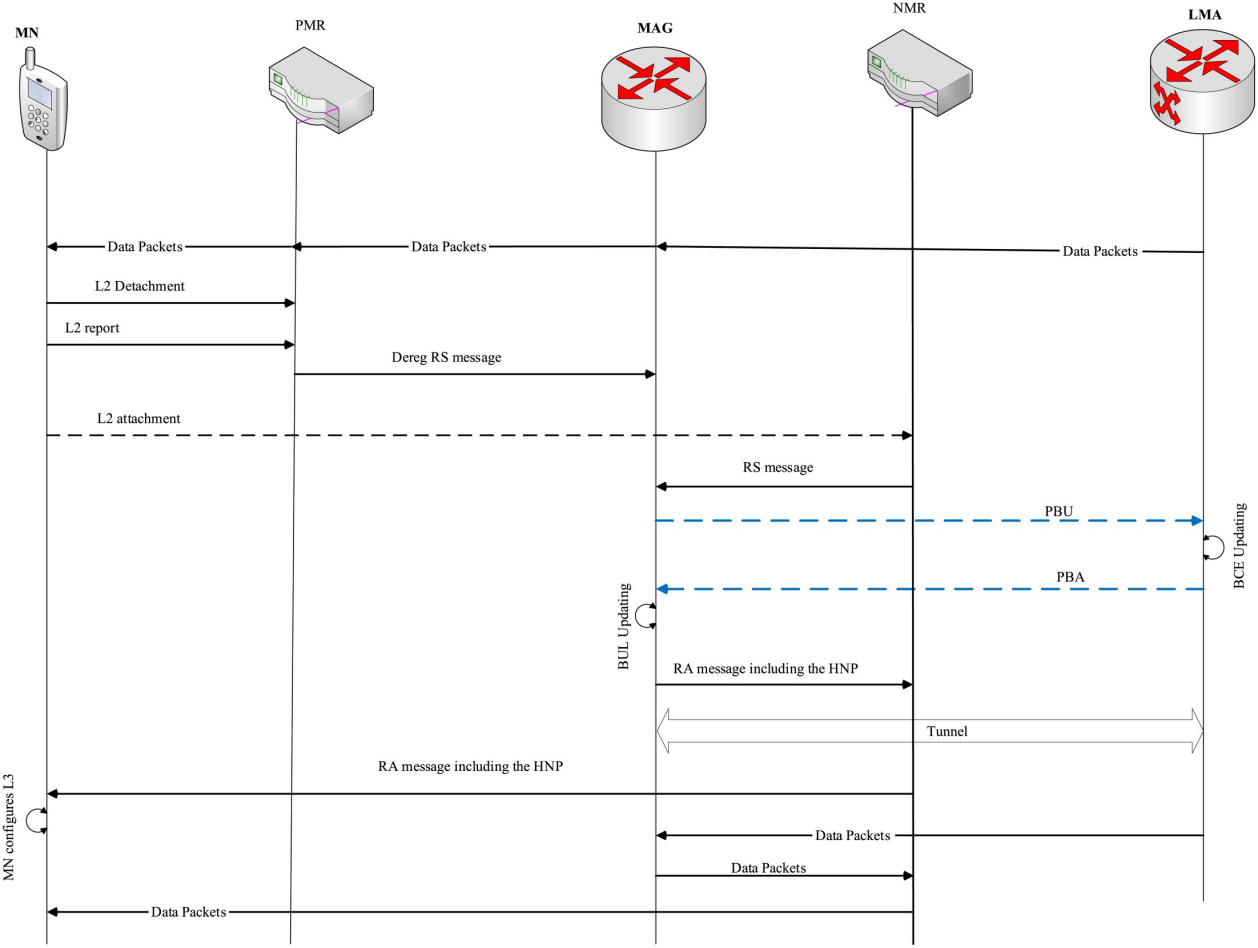
E-PMIPv6 registration process.

The aforementioned protocols support mobility either for individual MNs or for an entire mobile network. These protocols do not consider exempting MN from signaling handoff when moving from MAG to MR or from MR to a MAG. For that, an extension NEMO-enabled PMIPv6 (N-PMIPv6) [21] is proposed to provide network-wide mobility support while exempting the MN from the signaling handoff when the MN moves from an MR to MAG. The study in [22] considers three MNs’ handoff scenarios in order to support the mobility for the MNs or for the mobile network. The work in [12] considers the movement of the MNs between two MAGs, two MRs, from MR to a MAG or from the MAG to an MR during performing intra-handoff. This work is considered as a benchmark for this research work due to its considering the buffering cost and also all the possible handoff scenarios. Network-based NEtwork MObility supporting scheme (N-NEMO) and E-PMIPv6 are proposed to consider several scenarios of MNs movements during the handoff process (e.g., MAG-MAG, MR-MR, MR-MAG and MAG-MR) [12, 23]. In the N-NEMO and the aforementioned protocols, the packet loss is overlooked. For that the E-PMIPv6 scheme is developed in order to provide an efficient mobility scheme by eliminating the packet loss. In addition, the E-PMIPv6 scheme differs from other protocols with relation to BCEs utilization as it assigns only occupying one BCE for several MNs at the LMA. This is done through creating one BCE for MR to keep all the HNPs in the HNP field for all MNs attached to the MR. To fully understand the MR-enabled registration processes, the next paragraph discusses the registration processes of MR-enabled, as shown in Fig 1. These processes are performed as follows.

Upon entering the MR-enabled vehicle E-PMIPv6 domain, a Router Solicitation (RS) message is sent by the MR-enabled to the related MAG requiring HNPs for its MNs members. The MAG, upon receiving the RS message, creates a Binding Update List (BUL) for the MR and sends a Proxy Binding Update (PBU) message to the related LMA. In order for E-PMIPv6 to maintain the compatibility with the PMIPv6 registration process, the LMA in the E-PMIPv6 have to create a BCE for every HNPs required by the MAG. The RS and Router Advertisement (RA) messages are extended to be able to carry several prefixes. The fields of PBU and Proxy Binding Acknowledgment (PBA) messages, such as HNP option and number of HNP options used to determine the number of HNPs, are utilized. When LMA receives the PBU, it creates one BCE for the MR and setups a bi-directional tunnel with the related MAG in case there is no a bi-directional tunnel between them. Then, the LMA sends a PBU message to the MAG including the number of located prefixes. The HNP options field in the MR BCE is used to keep the located prefixes in a list in order to utilize the buffer resource. The MAG updates its BUL immediately upon receiving the PBA sent by the related LMA. Similarly to the LMA, the MAG fills the located prefixes in the HNP field in the MR BUL as a list. Therefore, the MAG sends RA message to the MR including the located prefixes. Then, when the MR receives the RA message, a distribution is performed to deliver each MN its prefix. Accordingly, when a new MN is attached to MR-enabled, the process of the MN registration process proceeds similarly to the registration process followed in the PMIPv6 protocol except that the LMA and the MAG do not need to create a BCE or BUL for the new attached MN. The located prefix of the attached MN is only added to the HNP list in the corresponding MR’s BCE in the LMA and MR’s BUL in the MAG. In case of MN detachment, the LMA and the MAG release the MN’s HNP from the BCE and BUL of the corresponding MR-enabled at LMA and MAG.

But despite the improvements made by the E-PMIPv6 scheme, the buffer resources utilization and lower signaling are dependent on the availability of MRs in the network. Consequently, when the network is not equipped with MRs or even when equipped with a small number of MRs, the enhancements become non-beneficial. To address this issue, this paper proposes a new scheme to provide an efficient handoff under different handoff scenarios. The new scheme enhances the system performance by reducing the handoff latency, buffering cost and signaling overhead. Moreover, the BCEs at the LMA are utilized more effectively.

## System model

**Fig 2:**
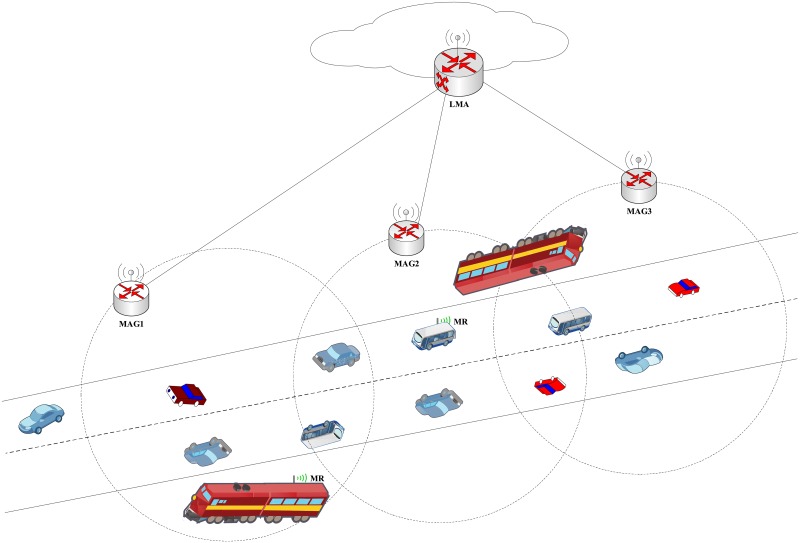
System architecture.

In order to show the effectiveness and efficiency of the AE-PMIPv6 scheme in urban vehicular networks, an infrastructure-based vehicular networks within a localized mobility protocol (PMIPv6) is used in this paper. As shown in Fig 2, the proposed infrastructure comprises one LMA, multiple MAGs and several MNs traveling within private and public transportations deployed along the roads in the city. The transportations (public and private) in the used infrastructure are classified into two parts according to whether they are equipped with MRs or not. In general, unlike buses and trains, which may be equipped with MRs to access the Internet, private vehicle are rarely equipped with MRs. The MR has two interfaces, one is to connect the MR with the MAG and the other one is to connect the MR with the attached MNs, in order to either forward or receive the MNs’ packets.

## The proposed scheme

An in-depth analysis of the E-PMIPv6 scheme within the localized mobility protocol, it shown that the scheme relies on the MRs to improve the system performance in terms of handover latency, buffer consumption and signaling overhead. Hence, the efficiency of the system is damaged by the absence of such MRs. To achieve this goal and in order to provide an efficient system, a virtual addressing and pool addressing mechanisms are employed in the proposed AE-PMIPv6 scheme. In this paper, the proposed AE-PMIPv6 scheme allows the system to merge the MNs’ binding information that are attached to the MAGs at specified time even when the MRs are absent. The specified time depends on the MAG configuration. This is performed in order to utilize the BCE at LMA efficiently. In addition, an addressing pool is employed to create HNPs in prior to MNs registration in order to improve the mobility management in terms of signaling cost and handover latency. The virtual addressing mechanism reduces the number of BCEs at the LMA by grouping the MNs and making the LMA deal with these MNs as one single unit. By grouping the MNs’ binding, the buffer resources are fully utilized and at the same time the addressing pool leads to lower the signaling overhead. The virtual addresses that are used in the system are obtained from the LMA according to the MAGs request. The virtual addresses are required before the MNs attachment to the MAGs in order to merge the attached MNs information inside the BUL of the MAGs based on their arrival time. Moreover, the MAG addressing pool keeps the HNPs that are required in advance in its pool in order to allocate them to the attached MNs as fast as possible.

In order to establish a session connection between MR/MN with the CN within the AE-PMIPv6 domain, the binding registration messages should be conducted by the MR/MN in order to acquire a HNP and then configure its IP address according to the obtained HNP. The initial binding registration and the handoff scenarios in AE-PMIPv6 scheme are explained in the next subsections.

### AE-PMIPv6 binding registration

**Fig 3:**
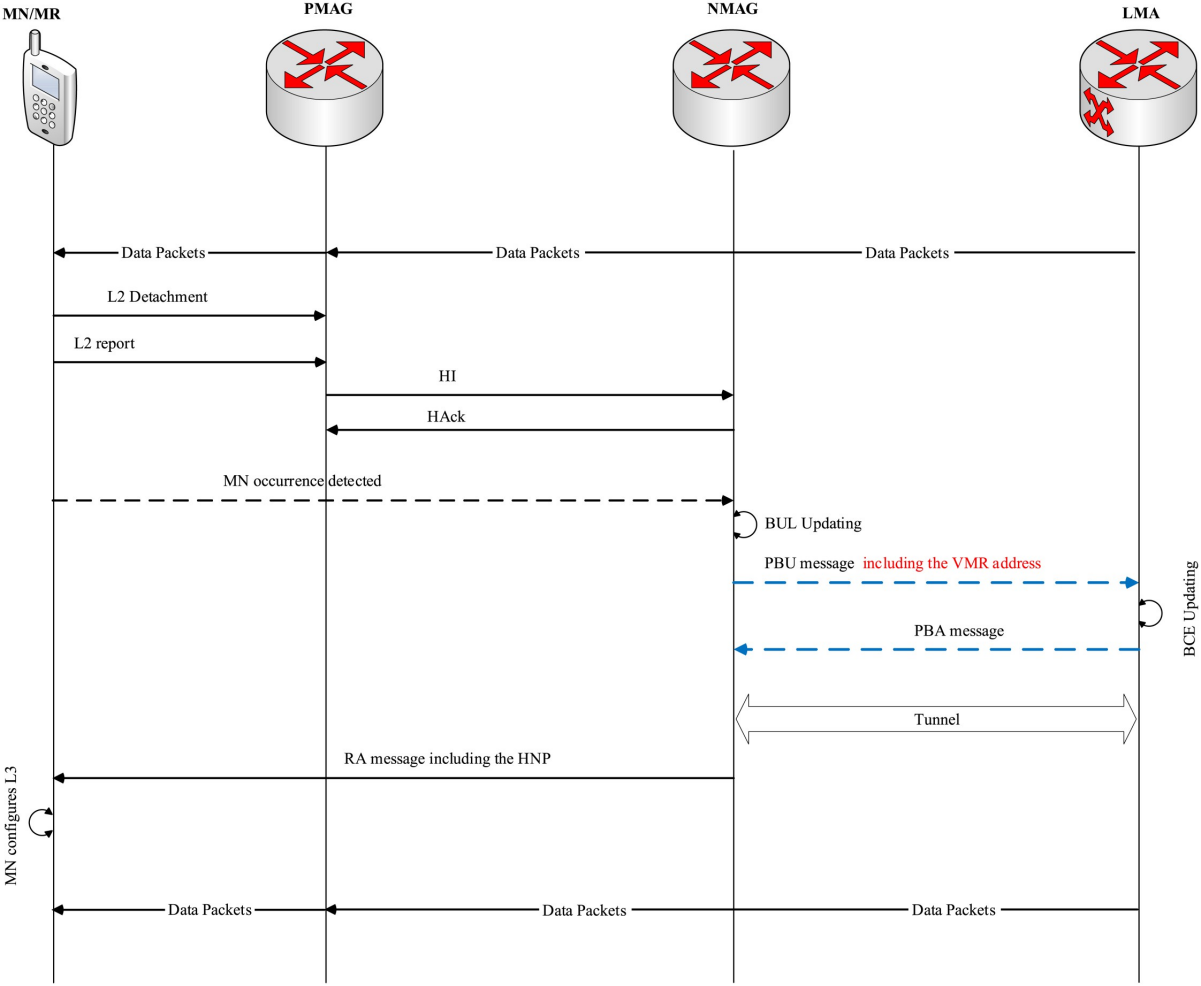
Registration process in AE-PMIPv6 scheme.

In urban vehicular networks, most of the private cars and the buses are not equipped with an MR. For that reason, each MAG in the AE-PMIPv6 domain requires multiple virtual addresses when attached to the LMA. These virtual addresses are deployed as virtual MRs to group the MNs/MRs connections at the MAG based on their attachment time. When the MAG receives the MN/MR connection, it sends a PBU message to the LMA to solicit an HNP for the attached MN in case there is no HNP in the MAG addressing pool, as shown in Fig 3. The HNP option field and a number of HNP options are utilized to indicate the number of the required HNPs in the case of MR attachment. The MAGs have benefited from grouping the MNs based on their arrival time, by reducing the searching time to find the BUL of the MN instead of searching in the whole records, especially when the number of MNs is very large. The registration signaling of the MN/MR is performed in the AE-PMIPv6 scheme as follows.

**Fig 4:**
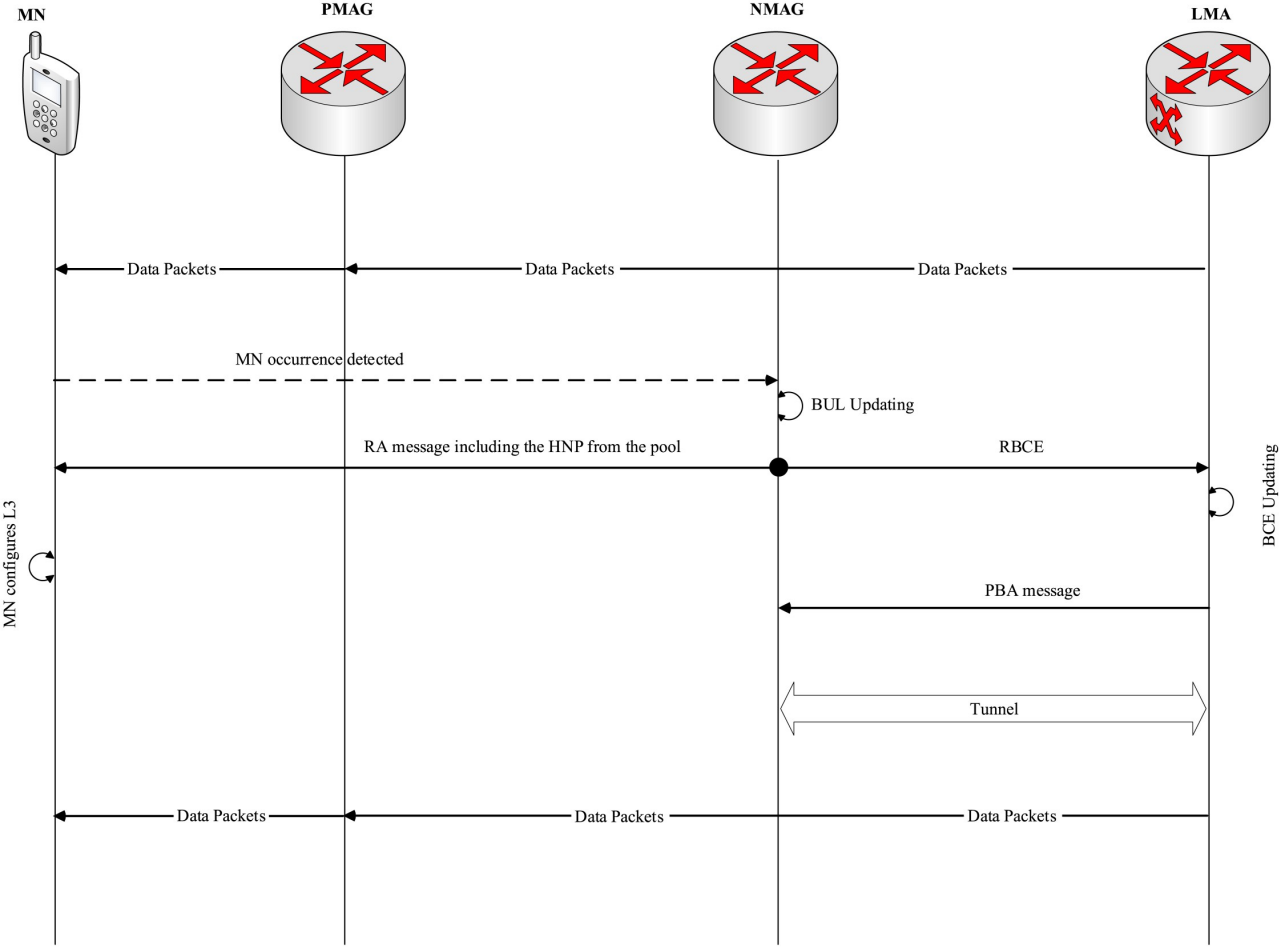
BUL at the MAG.

Whenever the MN/MR wishes to connect themselves to the MAG, RS message is sent by MN/MR to the MAG. Since the MAG has already created a BUL for every virtual address that has been required in advance, there is no need to create another BUL. Consequently, upon receiving the RS message, the MAG selects one of the virtual addresses stored in its pool and uses it as a virtual MR (VMR) for a limited time. The essence of the virtual MR is to delude the LMA that the connected MN/MR is coming from a real MR that is connected with it. Then, the MAG updates its BUL by adding the MN/MR information under the VMR record, as shown in Fig 4. Subsequently, the MAG sends a PBU including the MN/MR information to the LMA. These changes are recorded in the MAG’s BUL to guarantee delivering packets to the right MN/MR, as shown in Fig 4. Furthermore, the LMA upon receiving the PBU, updates the VMR BCE by adding the created HNP/HNPs to the HNP field. Then, the LMA sends a PBA message, including the HNP/HNPs to the corresponding MAG. Accordingly, the MAG, upon receiving the PBA, sends the allocated prefixes to the MN/MR to configure their IP address. Finally, the MR distributes the received allocated prefixes to the connected MNs as their HNPs in order to configure their IP addresses.

**Fig 5:**
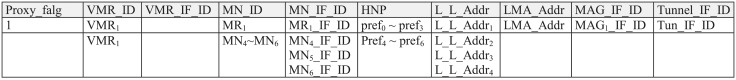
AE-PMIPv6 registration signaling using addressing pool mechanism.

As shown in Fig 5, the addressing pool scheme is used in the registration signaling in order to reduce the signaling cost and handover latency. The MAG in AE-PMIPv6 requires HNPs from the LMA in prior through sending a PBU message containing the required HNPs number and subsequently, the LMA creates the required HNPs. Then, the LMA sends the HNPs through the reply PBA message to the MAG. A new flag is added to the structure of PBU and PBA messages to distinguish them from the normal PBU and PBA messages. After receiving the PBA message, the MAG keeps the HNPs in its pool. These HNPs will be used in the future for the new attached MNs in order to reduce the handover time and signaling cost by eliminating the HNPs, PBA and PBU creation time. Creating several HNPs using one PBU and one PBA in advance significantly reduces the signaling cost. Therefore, the fields in PBA and PBU utilized need to be compatible for carrying several HNPs. Consequently, the registration of new MN is performed as follows.

Whenever a MN is detected to join the mobility domain, the MN sends an RS message to the MAG. Upon receiving the RS message sent from MN, the MAG searches its pool looking for an available address. Subsequently, the MAG sends the available address to the MNs through the RA message and at the same time it sends a request for updating the BCE (UBCE) to the LMA in order to update its BCE. Accordingly, the LMA updates its BCE and sends a PBA message including the HNP as well as establishing a bi-directional tunnel with the MAG. The MN configures its IP address in order to be able to send and receive the packets, when it successfully receives the RA message sent by the MAG.

**Fig 6:**
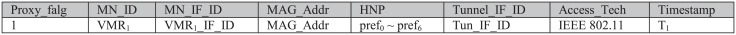
BCE at the LMA.

Unlike the E-PMIPv6 scheme, the AE-PMIPv6 maintains one BCE at the LMA for all MNs/MRs that arrive almost within the same time even when there is no MR in the network. This is done by creating one BCE for every VMR at the LMA, and the VMR’s HNP field in the BCE is used to store the required HNPs in a list. For example, the MR_1_ and its members MN_1_, MN_2_ and MN_3_ wish to attach the MAG and MN_4_, MN_5_, and MN_6_ that do not belong to MR_1_ wish to attach the same MAG almost at the same time. When the MAG receives the RS message of MR_1_, the MAG adds its information to one of the created VMR’s BUL, as shown in Fig 4. Then, the MAG sends a PBU message to the LMA asking LMA to register them. Accordingly, the LMA creates the HNPs (HNP_0_, HNP_1_, HNP_2_, HNP_3_) and includes them in the PBA message in order to send them to the corresponding MAG. One BCE is already created before for the VMR and consequently the registered MR is added to this BCE by adding the created HNPs in the HNP option field as a list, as shown in Fig 6. MN_4_ MN_5_ and MN_6_ send RSs to the MAG in order to start their registration. Then, the MAG employs the VMR to group the MNs that arrive almost at the same time of that MR. Instead of creating BULs separately for every arrived MN, the MAG adds their information to the VMR’BUL created in prior, as shown in Fig 4. Then, the MAG sends a PBU to the LMA in order to register the arrived new MNs. Consequently, the LMA upon receiving the PBU sent by the MAG, searches in its BCEs, hence the VMR has BCE. Accordingly, the LMA creates the required HNPs and adds them to the list in the HNP options field of the VMR’s BCE, which means there is no need for creating a new BCE or BUL for the new attached MNs. Then, the LMA replies to the MAG by sending a PBA message including the HNPs. Once receiving the PBA message, the MAG keeps the HNPs in the HNP options field. This scheme improves the buffer utilization by reducing data redundancy inside the BCE and BUL, especially when the LMA is responsible for registering thousands of nodes. Extra fields are added to the structure of BUL and BCE in order to enable the operation of the proposed AE-PMIPv6 scheme, as shown in Figs 4 and 6. To fully understand the data structure of BCE and BUL fields see [5].

**Fig 7:**
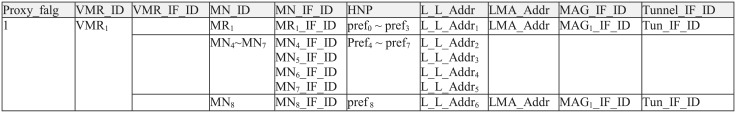
The updated BUL at the MAG.

**Fig 8:**
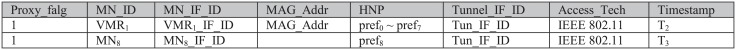
The updated BCE at the LMA.

After creating a BCE and BUL at the LMA and the MAG respectively, it is possible that new MNs/MRs will attach themselves to the MAG almost within the VMR time period. In such a case, the MAG upon receiving the RS messages, reacts to these messages according to the AE-PMIPv6 functionalities by adding them to the VMR address and sending PBU messages to the LMA, which in turn creates HNPs and adds them to the VMR HNP option list. The MNs move randomly in urban vehicular networks, so they can arrive to the network either simultaneously or at various times. For example, the MN_7_ is joined to the MAG within the VMR_1_ specified time, while MN_8_ is joined to the MAG after the VMR_1_ specified time is over. As shown in Figs 7 and 8 respectively, the MN_7_ is added to the BUL of VMR_1_ in the MAG and to the BCE of VMR_1_ at the LMA. This is because the VMR_1_ already has information (BUL and BCE) at the MAG and LMA, which eliminates the need for creating BUL and BCE in both MAG and LMA. In the case of MN_8_ after the VMR_1_ has finished its time, a new BUL and BCE for the MN_8_ must be created in both MAG and LMA, as shown in Figs 7 and 8.

### AE-PMIPv6 handoff signaling process

The AE-PMIPv6 handoff signaling considers the handoff either between MAGs, MRs or between a MAG and MR or vice versa. In this section, only two cases of handoff are demonstrated to explain the usage of the virtual addresses mechanism.

**Fig 9:**
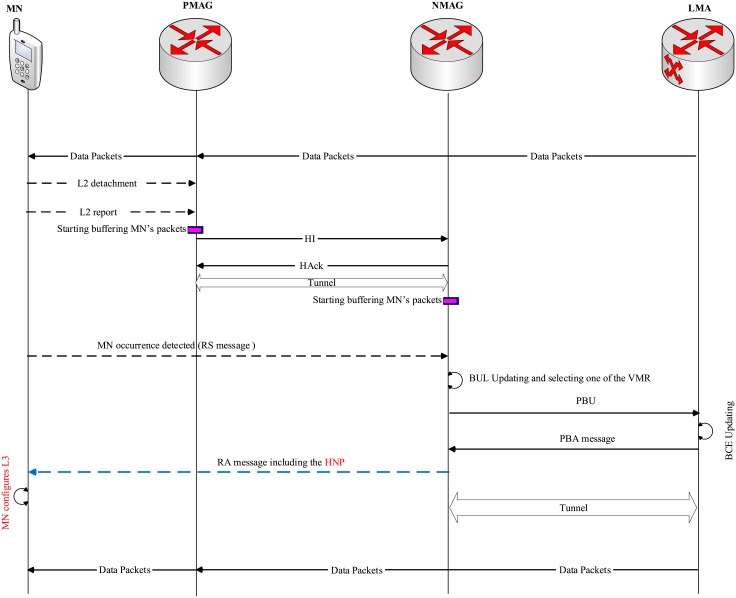
The MN handoff signaling in the AE-PMIPv6 scheme.

In case of MN handoff, the handoff occurs when the MN or MR changes its point of attachment(across different networks), as shown in Fig 9. In the MN handoff case, upon detecting L2 detachment, the MN sends a report to the PMAG including the MN Identifier (MN-ID) and the NMAG-ID. A bi-directional tunnel is established by the PMAG by exchanging HI and HAck messages. After realizing the MN attachment by the NMAG, the NMAG sends a PBU message to the LMA in order to update the binding information. Moreover, the PMAG starts buffering the packets instead of forwarding them to the MN in order to prevent packet loss. The MAG sends a PBU message, including the MN-ID, VMR-ID, MAG-ID and all other necessary information, to the related LMA. Upon receiving the PBU, the LMA creates the MN’s HNP and adds it to the new BCE of the new VMR after removing it from the old VMR belonging to the old MAG. In addition, the LMA sends PBA to the NMAG including the MN’s HNP. Finally, the NMAG, upon receiving the PBA, sends the HNP to the attached MN in order to complete the handoff process.

**Fig 10:**
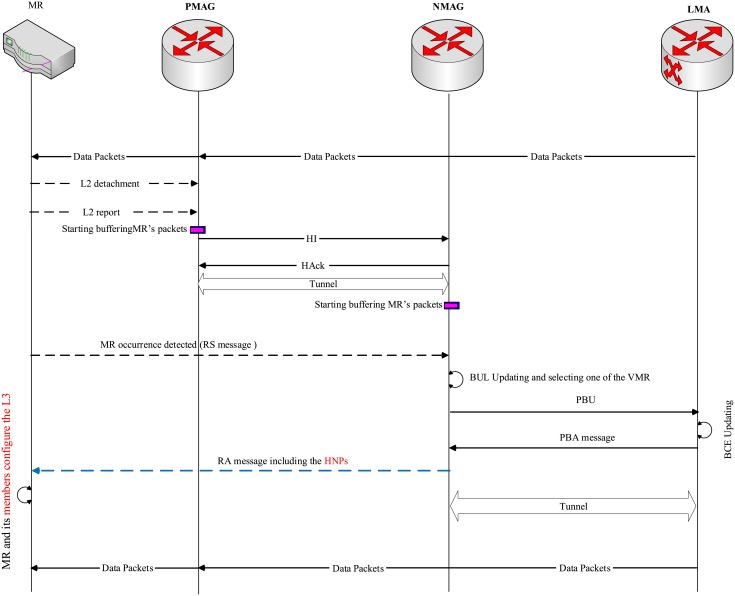
The MR handoff signaling in the AE-PMIPv6 scheme.

In case of MR handoff, the handoff process is triggered when the MR moves to another MAG, as shown in Fig 10. The signaling process is similar to the handoff scenario mentioned earlier. L2 report (MR-ID, MN-IDs and Previous MR(PMR)) is sent to the NMAG by the MR when the L2 detachment is detected. The NMAG, upon realizing this attachment through receiving the RS message sent by the MR, sends a PBU to its related LMA including the necessary information about the MR and its members. Then, the LMA moves the HNPs of the MR and its member from its old BCE and adds them to the list of the HNP field options of the new VMR that belongs to the related MAG. A PBA reply message is sent by the LMA to the related MAG including the HNPs. The MAG then sends an RA to the MR including the HNPs. Finally, the MR and its members have the capability to restart their communications without any change in their IP stack. For an in-depth overview on the handoff processes, which are performed by the E-PMIPv6 scheme (e.g., MR-MR, MAG-MAG, MAG-MR and MR-MAG) see [12].

## Performance evaluation

To study the performance of the proposed AE-PMIPv6 in terms of buffering cost, an analytical analysis is performed in this section. In order to get an identical platform for comparative purpose, the related assumptions and the notations that are used in [12] are reused in this work and are listed below. Due to the buffer resource limitation, the buffer resource utilization is an important performance metric that is used to measure the buffering cost. In the AE-PMIPv6 domain, the MNs information is kept in the BCE of the LMA. As observed from Figs 6 and 8, one BCE has the ability to store the binding information of MRs and MNs. As illustrated in Table 1, the *L*_*BCE*_ and the *L*_*HNP*_ represent the length of the BCE and HNP of the MN respectively. For the sake of the simplicity, one scenario is considered in this analysis to show the effect of the proposed AE-PMIPv6 scheme on the buffering cost. This scenario occurs when the handoff is performed by both MRs and MNs within the LMA domain regardless of their handoff time. In this scenario, the handoff MR has no member and the MNs come from MAG or from another MRs. Table 2 presents the number of MNs and the number of MRs used for the analyses. Consequently, the buffering cost of keeping information of MNs and MRs in the BCEs at the LMA in the AE-PMIPv6 scheme can be mathematically expressed as:

$$\begin{array}{c} BC_{LMA}^{BCE}=\varepsilon_{LMA}\left(.N_{MR\in LMA}^{*}.\left(N_{SMN}^{*}.L_{BCE}\right)\right)+\varepsilon_{LMA}.\left(N_{VMR}.\left[\sum_{i=0}^{1}Y_{i}+L_{BCE}\right]\right) \end{array}$$ (1)

where *Y*_*i*_ = *n*_*i*_.*y*_*i*_, *i* represents the type of connected node (MR or/and MN) at LMA and MAG. The *N*_*MR*∈*LMA*_ and $N_{MR\in LMA}^{*}$ are denoted to the numbers of MR enabled vehicles and MR disabled vehicles at the LMA domain, respectively and *N*_*MNs*_ and *N*_*MRs*_ are representing the average number of the handoff MRs/MNs on a VMR and the $N_{SMNs}^{*}$ are denoted to the average number of MNs on an MR disabled vehicle.

**Table 1:** Notations and settings.

Parameter	Value	Description
*L*_*PBU*_	72	Length of PBU message (byte)
*L*_*PBA*_	72	Length of PBA message (byte)
*L*_*BCE*_	62	Length of BCE message (byte)
*L*_*BUL*_	62	Length of BUL message (byte)
*L*_*HNP*_	8	Length of HNP message (byte)
*ε*_*LMA*_	0.5	Represents the buffering factor at the LMA
*ε*_*MAG*_	0.5	Represents the buffering factor at the MAG
$N_{MR\in LMA}^{*}$	20	Represents the MR disabled vehicles at the LMA domain
$N_{SMN}^{*}$	4	Represents the number of MNs on MR disabled vehicle at the LMA domain
*N*_*VMR*∈*LMA*_	4	Represents the virtual MRs at the LMA domain
*N*_*MR*∈*LMA*_	3 ∼ 20	Number of Mobile Routers
*N*_*MNs*_	3 ∼ 30	Represents the MR enabled vehicles at the LMA domain

**Table 2:** Parameter values obtained from Eq 1.

*n*_0_	*n*_1_	*y*_0_	*y*_1_	*n*_0_.*y*_0_	*n*_1_.*y*_1_	*n*_0_.*y*_0_ + *n*_1_.*y*_1_	*β*
5	3	8	8	40	24	64	126
5	4	8	8	40	32	72	134
7	6	8	8	56	48	104	166
10	8	8	8	80	64	160	206
12	10	8	8	96	80	176	238
15	12	8	8	120	96	216	278
17	14	8	8	136	112	248	310
20	16	8	8	160	128	288	350
25	18	8	8	200	144	344	406
30	20	8	8	240	160	400	462

Then the final relation will be written as:

$$\begin{array}{c} BC_{LMA}^{BCE}=\varepsilon_{LMA}.N_{MR\in LMA}^{*}.N_{SMN}^{*}.L_{BCE}+\varepsilon_{LMA}.\left(N_{VMR\in LMA}.\left[y_{0}.n_{0}+y_{1}.n_{1}+L_{BCE}\right]\right) \end{array}$$ (2)

where

*n*_0_ = *N*_*MNs*_,

*n*_1_ = *MRs*,

*y*_0_ = *L*_*HNP*_ of MN,

*y*_1_ = *L*_*HNP*_ of MR,

*y*_0_.*n*_0_ + *y*_1_.*n*_1_ + *L*_*BCE*_ = *β*.

Similarly, the MNs’ binding information must be maintained at specific MAG. *L*_*BUL*_ is denoted to the length of the BUL of a MN at a MAG, and the buffering cost for keeping BULs at a MAG is

$$\begin{array}{c} BC_{LMA}^{BCE}=\varepsilon_{MAG}.\left(N_{MR\in MAG}^{*}.\left(N_{SMN}^{*}.L_{BUL}\right)\right)+\varepsilon_{MAG}.\left(N_{VMR\in MAG}.\left[\sum_{i=0}^{1}Y_{i}+L_{BUL}\right]\right) \end{array}$$ (3)

Where

*y*_0_.*n*_0_ + *y*_1_.*n*_1_ + *L*_*BUL*_ = *ω*.

**Fig 11:**
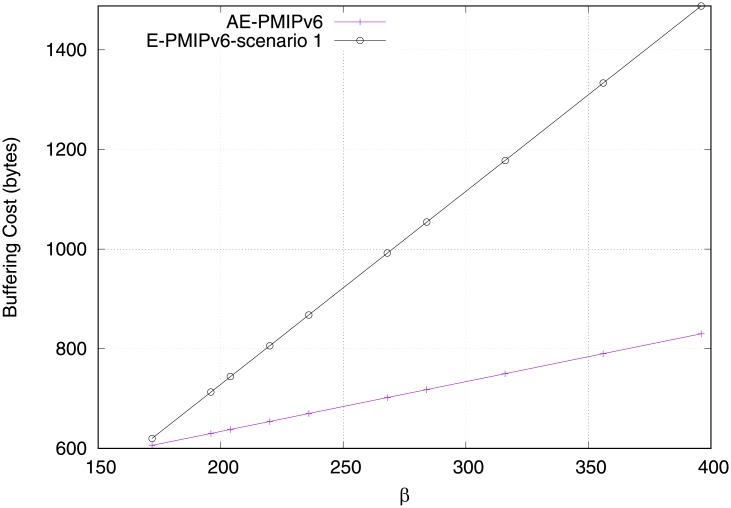
Buffering cost at the LMA.

As shown in Fig 11, a comparison is performed between the AE-PMIPv6 and E-PMIPv6 schemes. From the figure, it can be observed that the buffering cost of the E-PMIPv6 scheme increases rapidly as a function of the number of the MNs and the MRs compared to the proposed AE-PMIPv6 scheme. This is because the LMA in the E-PMIPv6 domain has to create a BCE for every connected MR. In addition, the LMA in the E-PMIPv6 domain have to create a separate BCE for each MN moving from one MAG to another individually in order to maintain such moving nodes’ information. However, the proposed AE-PMIPv6 scheme only creates one BCE for both connected MRs and MNs regardless of their handoff time, and only the HNP field at the BCE of the VMR have to be extended to keep the allocated HNPs of the connected MNs/MRs, which significantly reduces the occupied buffer space at LMA, as shown in Fig 8.

**Fig 12:**
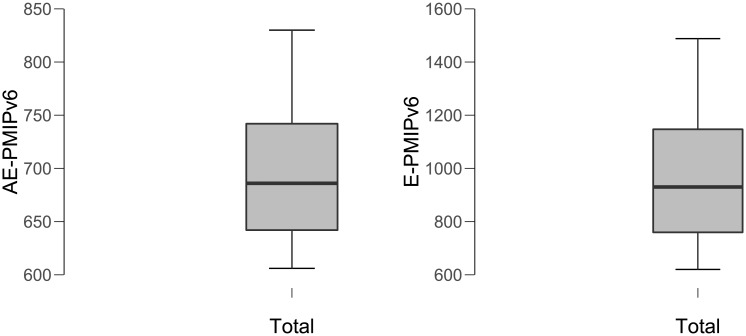
The mean and standard deviation of AE-PMIPv6 and E-PMIPv6 schemes.

In order to show the obtained data’s significant performance of the proposed scheme, the descriptive statistics (e.g., mean and the standard) are calculated, as shown in Table 3. According to Fig 12, we clearly observe that the average value and standard deviation of AE-PMIPV6 scheme buffering cost are less than the E-PMIPv6 scheme.

**Table 3:** Descriptive statistics.

	*AE*—*PMIPv*6	*E*—*PMIPv*6
Mean	698.8	979.6
Std. Deviation	73.34	284.2

## Simulation and experiments

For the purpose of evaluating the AE-PMIPv6 scheme under PMIPv6 environment, Network Simulator version 2 (NS2) based PMIPv6 package is downloaded [24]. Then, this package is extended to implement the proposed AE-PMIPv6 scheme and then compared to the E-PMIPv6 scheme in terms of the cost of the buffering, signaling and the handoff NS2 [25]. This is to ensure that the AE-PMIPv6 scheme has achieved its main goal, which is to enable the buffer resource utilization, and to guarantee low handover latency and low signaling cost whether the network is equipped with MRs or not.

The work in [12] is re-implemented and considered in order to validate the efficiency of the proposed AE-PMIPv6.

### Simulation setup

To further analyze the performance of the proposed AE-PMIPv6 scheme, a simulation is setup using the network simulator version 29 (ns-2.29) with the National Institute of Standards and Technology(NIST) package running on opensuse Linux version 13.2. The opensuse operating system an on Intel machine with a 3.20 GHz Core i5-3470 and 8GB of RAM. The file tracing, extraction, and text processing are performed using AWK scripting language. The Gnuplot graphics tools version 5.0 is used to generate the results. The simulation experiments are carried out using various MN numbers that move in a straight line with a cyclic lane to enter the overlapping area between the MAGs. In the simulation environment, the MAGs are distributed along two opposite roads and the vehicles that equipped with the MNs are traveling on straight roads. Every MAG has an overlapping area with its neighboring MAGs in order to create an overlapping coverage area to allow the MN to continuously receives traffic, while it follows the deterministic path and incurs a handover among several MAGs. The distance between two MAGs is about 400 meters. Table 4 shows the important parameter associated with their values that are used in the simulation parameters where lengths of messages L2 report, Dereg RS, RS, PBU, PBA, RA, HI and HAck are represented by *L*_*l*2_, $L_{D_{R}S}$, *L*_*RS*_, *L*_*PBU*_, *L*_*PBA*_, *L*_*RA*_, *L*_*HI*_, and *L*_*HAck*_, receptively.

**Table 4:** Simulation parameters.

Parameters	Type	Values
UDP:	Traffic type	CBR
	Packet size	512 bytes
	Interval	0.1 sec
IEEE 802.11:	MAC bandwidth	2Mb/s
	Transmission range	250m
	Overlapping range	100m
	Radio-propagation model	TwoRayGround
	Topography area	970m × 970m
	Specific topography area (e.g., Handoff experiments)	970m (width) × 2970m (Hight)
Wired Link(rate/delay):	Between CN & LMA	10Mbps/11ms
	Between AR & MAG	100Mbps/1.7 ms
	Between LMA & MAG	100Mbps/1.3 ms
	Between MN & AR	11Mbps/10 ms
	Between MN & MAG	11Mbps/10 ms
Antenna model	Antenna/OmniAntenna	-
Mobile Node:	Velocity	5 ∼ 30 m/s
	Mobility pattern	Straight line
*N*_*MNs*_	-	5 ∼ 20
$N_{SMN}^{*}$	-	0 ∼ 4
*L*_*DATA*_	-	512 bytes
MRs	-	0 ∼ 3
MAGs	-	4
LMA	-	1
*L*_*l*2_	-	52 bytes
*L*_*D*−*RS*_	-	52 bytes
*L*_*RS*_	-	52 bytes
*L*_*PBU*_	-	72 bytes
*L*_*PBA*_	-	72 bytes
*L*_*RA*_	-	92 bytes
*L*_*HI*_	-	52 bytes
*L*_*HAck*_	-	52 bytes
*L*_*BCE*_	-	62 bytes
*L*_*BUL*_	-	62 bytes
*L*_*HNP*_	-	8 bytes
Time:	Simulation end	100 sec

### Results and discussion

In this section, we report and discuss the evaluation results conducted based on two different scenarios, namely, on whether the network is equipped with MRs or not. We have applied the virtual addressing mechanism and the virtual addressing with the addressing pool mechanisms separately in both scenarios of E-PMIPv6 scheme, as depicted in the results. In order to obtain statistically reliable results, we have run each experiment 50 times with different seeds and reported the average results of such experiments.

**Fig 13:**
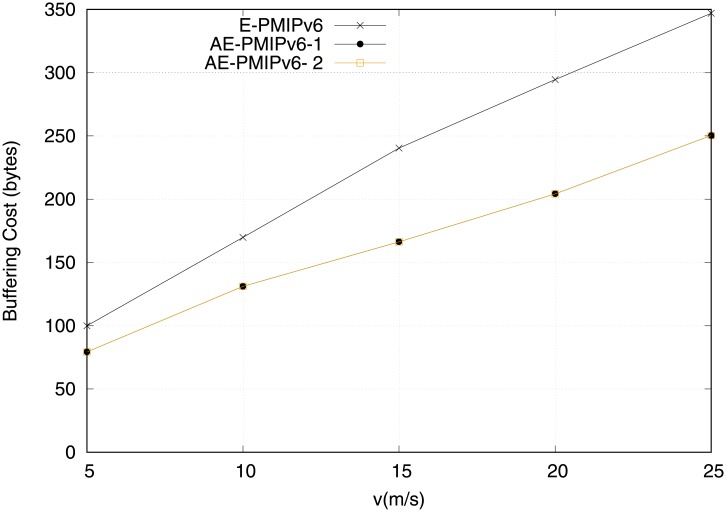
Buffering cost obtained from scenario 1.

We started this evaluation by inspecting the efficiency of both schemes (i.e. AE-PMIPv6, E-PMIPv6) in terms of buffer resource utilization is required during the handoff process. In fact, in context of mobility management there are two types of buffers used to maintain the information that facilitates handoff process; 1) Binding information buffers: those buffers are used to maintain the MNs’ binding information; 2) Packets Buffers: those buffers are used for temporarily buffering the packets during the MNs movements. Fig 13 measures the buffering cost with respect to the BCE and BUL space utilization. The AE-PMIPv6 scheme is compared with the E-PMIPv6 scheme under various vehicle speeds in this figure. It is clear from the figure that the buffering overhead increases as a function of vehicles speed. Considering the increase of MNs’ handoff, moving with high speed increases the chance of having a buffer space occupied by the handoff MNs. It also observed that the AE-PMIPv6 proposed scheme has a better buffering overhead compared to the E-PMIPv6 scheme. This is mainly attributed to the fact that the E-PMIPv6 scheme requires to maintain one BCE entry for the MRs and their members at both the LMA and the MAG. However, the number of BCEs that needs to be maintained in the proposed AE-PMIPv6 scheme is reduced as a result of grouping the MNs, even if they do not belong to the same MR. This is achieved by sharing the same virtual address, hence, requiring only one BCE per group rather than per MR as in the E-PMIPv6 scheme.

**Fig 14:**
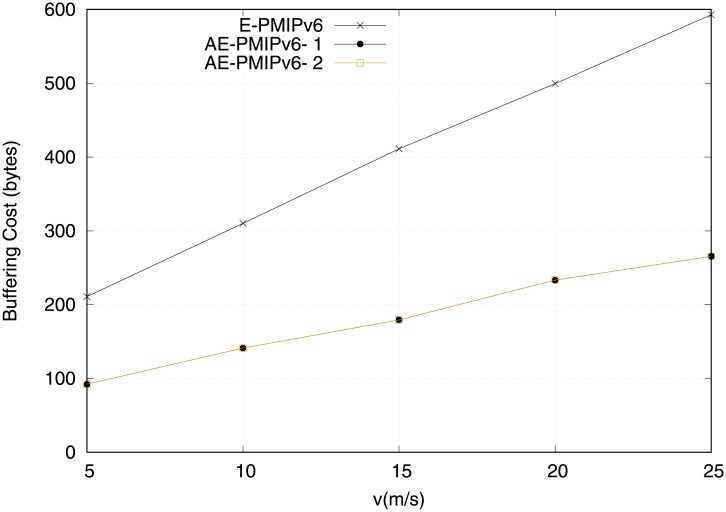
Buffering cost obtained from scenario 2.

The efficiency of the proposed AE-PMIPv6 and the E-PMIPv6 schemes in terms of buffering cost is shown in Fig 14. It is clearly observed that the E-PMIPv6 experiences the highest buffering cost due to the need to maintain a BCE for each MN. In addition, the buffering cost increases steadily in the E-PMIPv6 scheme when the number of the handoff MNs increases because the E-PMIPv6 ‘s buffering cost is fully dependent on MRs that exist in the network. The proposed AE-PMIPv6 in both scenarios (i.e. equipped or not equipped with MRs) achieves better performance compared to the E-PMIPv6 scheme. This achievement comes as a result of using virtual addressing that utilizes the buffer resources efficiently regardless of network infrastructure.

**Fig 15:**
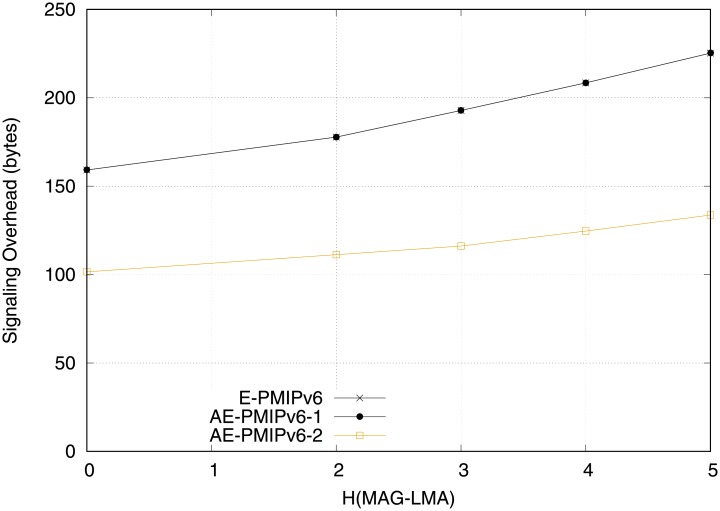
Signaling overhead obtained from scenario 1.

Signaling overhead, which greatly impacts the QoS by consuming the bandwidth and accordingly reducing the signaling, should be considered. Fig 15 compares both schemes in terms of signaling overhead in the first scenario (i.e. network equipped with MRs). As can be observed from the figure, both schemes show a similar performance even when AE-PMIPv6 scheme applies virtual addressing. This is because of the transparency of mobility-related signaling during the handoff process, which in turn alleviates the MNs from the concerns related to IP stack complexity. However, applying addressing pool in scenario 2 greatly reduces the signaling overhead. The superiority of the proposed AE-PMIPv6 scheme is attributed to the advanced creation of HNPs that leads to reduce the number of messages required to achieve the MNs’ handoff process.

**Fig 16:**
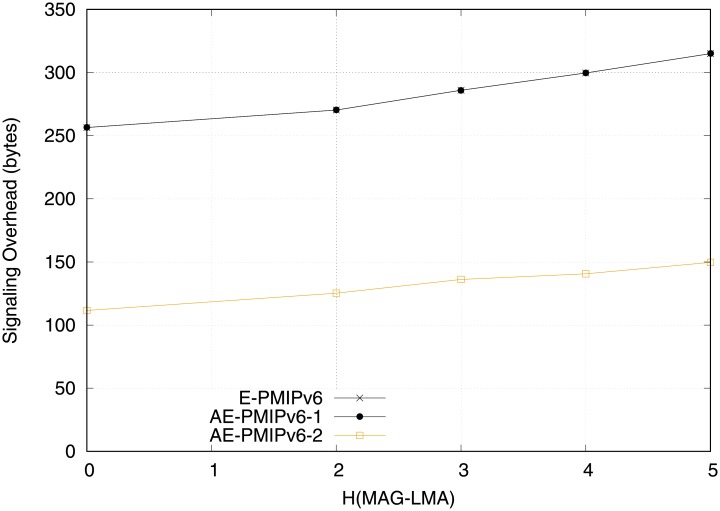
Signaling overhead obtained from scenario 2.

Fig 16 reveals the superiority of the proposed AE-PMIPv6 scheme compared to the E-PMIPv6 scheme in terms of signaling cost in the second scenario (i.e. when the network is not equipped with MRs). It is observed that the AE-PMIPv6 that applies addressing pool has a better signaling cost than the E-PMIPv6 and AE-PMIPv6 that employs the virtual address only. This is can be attributed to the advance creation of HNPs, which eliminates the PBU and PBA signaling during the MNs registration.

**Fig 17:**
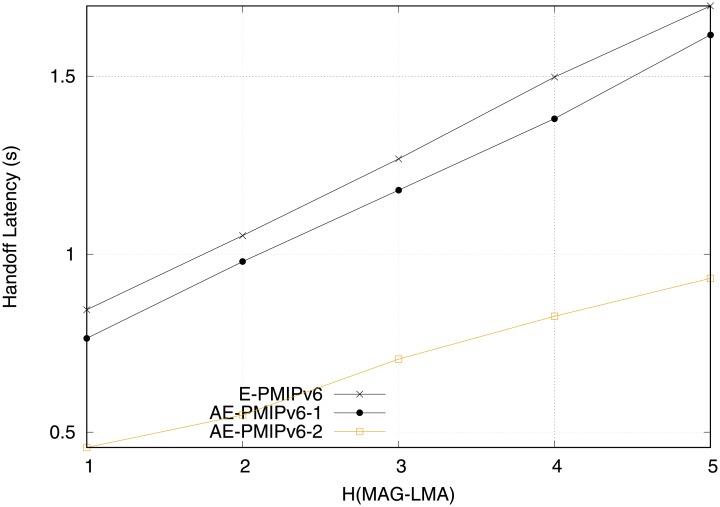
Handoff latency obtained from scenario 1.

Handoff latency is also an important factor in the mobility management that should be considered due to its effect on the system throughput. The handoff latency is defined as the time interval between *t*_1_ and *t*_2_. *t*_1_ is the time at which last packet is received by the handoff MN successfully from the PMAG. *t*_2_ is the time at which the first packet is received by the handoff MN successfully from the NMAG. Intuitively, as the number of the hops between the MAG and the LMA increases, the handoff time increases. Fig 17 shows a comparison of the handoff latency between the proposed AE-PMIPv6 and E-PMIPv6 schemes as a function of the number of hops between the LMA and the MAG. It is clear from the figure that the handoff latency of the E-PMIPv6 scheme is higher than that of the proposed AE-PMIPv6 in scenario 2. This is attributed to using the virtual addressing mechanism, which significantly reduces the time that is needed to pick up the MNs information from the BUL and BCE at the MAG and LMA respectively. Despite the fact that the buffer utilization is enhanced by using only one BCEs for each MR and its members, The proposed AE-PMIPv6 scheme shows a better buffer utilization compared to the E-PMIPv6 due to the notion of grouping the largest possible number of MNs’ binding information in one BCE, based on their arrival times. The AE-PMIPv6 scheme in scenario 2 registers the best performance in terms of handoff by eliminating the need for IP acquisition and the need to create the PBU and PBA messages, which is a time-consuming process during the MN handoff.

**Fig 18:**
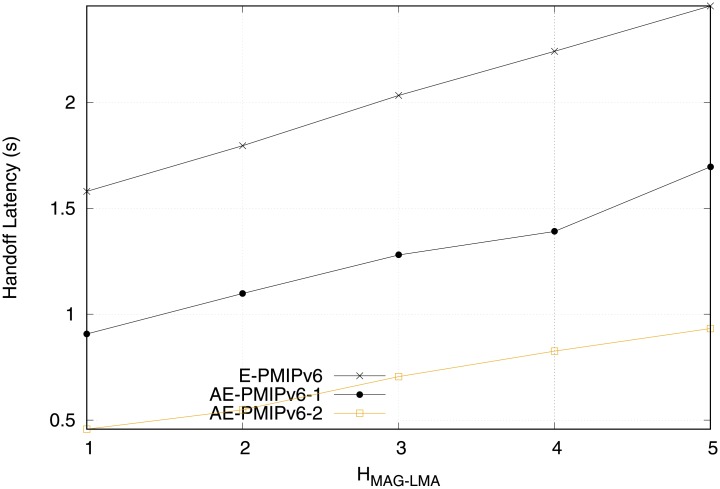
Handoff latency cost obtained from scenario 2.

Fig 18 shows the system performance behavior of the AE-PMIPv6 and E-PMIPv6 schemes when the network is not equipped with MRs in terms of handoff latency. It is observed that the E-PMIPv6 scheme experiences the highest handoff latency due to the absence of the buffer utilization mechanism. The AE-PMIPv6 with virtual addresses has improved the performance significantly compared to the E-PMIPv6 scheme. This is because the proposed scheme has the ability to utilize the buffer resources even when there is no MRs in the network. The proposed AE-PMIPv6 scheme in scenario 2 that applies the addressing pool mechanism outperforms the other schemes. This can be attributed to the elimination of the IP acquisition time from the handoff time, which greatly reduces the handover delay. Moreover, creating the HNPs in prior improves the performance through reducing the number of exchanged messages as each message require a specific time to be created such as PBU and PBA that are exchanged between the MAG and LMA to create HNPs for the new MNs.

## Conclusion

In this paper, an effective mobility management scheme has been proposed in order to provide a seamless handover for the MN in the urban vehicular network. By means of simulation, it has been shown that the developed scheme has the capacity to reduce the signaling cost and the buffering overhead in the network. The proposed AE-PMIPv6 scheme introduces the idea of employing the virtual addressing and addressing pool mechanisms during the registration phase of the MN providing the MAG with the capacity to merge the binding information of the MNs arriving almost simultaneously and to create HNPs in advance. In addition, the proposed scheme provided the MAG with the capacity of merging the information of such MNs into one BUL and also the LMA with the capacity of merging the required HNPs in a single BCE enabling a better resource utilization and less buffering cost. Finally, employing the addressing pool mechanism has enabled the proposed scheme to acquire the HNPs before the handoff/registration of MNs reducing both the signaling cost and the handoff latency. The simulation results have demonstrated that AE-PMIPv6 outperforms the E-PMIPv6 scheme in terms of buffering overhead, signaling cost and handoff latency.
